# A direct learning approach for detection of hotspots in microwave hyperthermia treatments

**DOI:** 10.1007/s11517-025-03343-9

**Published:** 2025-03-11

**Authors:** Hulusi Onal, Enes Girgin, Semih Doğu, Tuba Yilmaz, Mehmet Nuri Akinci

**Affiliations:** 1 Department of Electronics and Communication Engineering, Istanbul Technical University, Maslak, Istanbul, 34469 Turkey; 2 Burgan Bank, Maslak, Istanbul, 34485 Turkey

**Keywords:** Breast imaging, Deep learning, Hyperthermia, Microwave imaging, Temperature monitoring

## Abstract

**Abstract:**

This paper presents a computational study for detecting whether the temperature values of the breast tissues are exceeding a threshold using deep learning (DL) during microwave hyperthermia (MH) treatments. The proposed model has a deep convolutional encoder-decoder architecture, which gets differential scattered field data as input and gives an image showing the cells exceeding the threshold. The data are generated by an in-house data generator, which mimics temperature distribution in the MH problem. The model is also tested with real temperature distribution obtained from electromagnetic-thermal simulations performed in commercial software. The results show that the model reaches an average accuracy score of 0.959 and 0.939 under 40 dB and 30 dB signal-to-noise ratio (SNR), respectively. The results are also compared with the Born iterative method (BIM), which is combined with some different conventional regularization methods. The results show that the proposed DL model outperforms the conventional methods and reveals the strong regularization capabilities of the data-driven methods for temperature monitoring applications.

**Graphical abstract:**

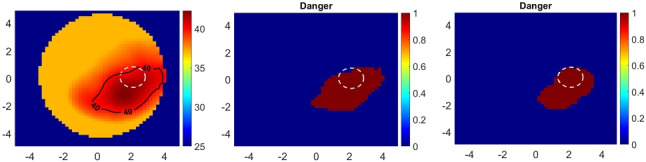

## Introduction

Microwave hyperthermia (MH) is a non-invasive cancer treatment technique that aims to expose the tumor area to relatively high temperatures, which are generally in the range of 40–45 $^\circ$C. MH can be used alone or in combination with treatment methods such as radiotherapy and chemotherapy [1–3]. It is very important that the healthy tissues stay at safe temperature values while the tumor area is heated during MH treatment [4]. Therefore, the microwave energy should be well focused on the tumor area [5]. During the treatment, it is a challenging task to achieve the desired temperature in the regions with a non-invasive method and to monitor these temperatures at the same time. By using fiber optic catheters, which is an invasive method, temperature measurements can be performed during MH treatment [6]. However, it is very difficult to place the catheters on the patient, and it is also a painful process. It would be more appropriate to use non-invasive methods for the patient to have a more comfortable treatment process. Therefore, the development of methods such as magnetic resonance imaging (MRI), ultrasound (US) imaging, microwave radar-based imaging, and microwave tomographic imaging (MWT) is important for non-invasive temperature monitoring during hyperthermia treatments.

MRI is an effective and feasible method for hyperthermia treatment monitoring [7, 8]. However, this system is difficult to set up and quite costly. US imaging is appealing in terms of availability and cost; however, motion artifacts may dominate the small signal changes connected to the apparent tissue displacements brought on by temperature variation [9]. Another method for non-invasive temperature monitoring is microwave radar-based imaging [10]. The radar-based methods are generally computationally cheap; nevertheless, their capabilities decrease in inhomogeneous media. MWT methods, which are generally based on the solution of the inverse scattering problem (ISP), have been utilized for quantitative [11, 12] and qualitative [13, 14] temperature monitoring. Even if the numerical and experimental studies show the feasibility of the MWT approaches, these methods still require knowledge of the internal total electric field over the computation domain to provide quantitative information for temperature distribution [11, 12].

Deep learning (DL) methods have also been utilized for solution of the ISPs to overcome the restrictions of the conventional methods. The DL methods come to the forefront due to their capabilities for providing real-time imaging and better regularization [15–18]. The success of the DL methods in the ISPs shows the potential of these methods for temperature monitoring applications. To the best of our knowledge, there is only one study [19] that uses DL for thermal treatment monitoring using microwaves. In [19], a two-stage process is applied to classify the heated status of the region of interest. Firstly, a conventional inversion scheme is applied using the distorted Born approximation and the truncated singular value decomposition. After that, the estimated dielectric properties (DPs) of the region are supplied to convolutional neural network (CNN) to find a DL model providing information on the heated status of each region of interest.

In this study, we propose a direct learning approach to detect the hotspots in MH treatments. The word “hotspot” means a cell (a pixel) in the imaging domain exceeding a dangerous temperature level. In contrast to [19], our DL model directly matches the input data to the output image; therefore, it avoids using any conventional inversion process. This is important to save the real-time imaging capabilities of DL methods and avoid the problem of regularization parameter selection in conventional inversion methods. The proposed model gives an image as output, which shows the hotspot distribution over the imaging domain in real time. The capability of providing a real-time image of the treatment region is important so that the medical staff can continuously follow the treatment process. Moreover, once the image is obtained, it is straightforward to decide the danger status, which indicates whether there is any hotspot arising on healthy tissues (the tissues outside the tumor). From a practical perspective, this makes it possible that the system can react in real time for patient safety without any visual inspection by the staff. An important contribution of the presented study is to overcome the problem of not being able to detect temperature changes on tissues with low dielectric values, which we mentioned in our previous study [11]. In this way, the accuracy in detecting hotspots increases significantly, demonstrating that data-driven methods have strong regularization capabilities in terms of the hyperthermia monitoring problem. Furthermore, the noise effect is also very important for the hyperthermia monitoring problem because the temperature dependency of DPs of tissues is very low at MH treatment temperatures [20, 21]. Therefore, we give a detailed discussion about the effect of noise on training and test data, for which there is a lack of investigation in the literature. It is also worth noting that the study was performed for breast hyperthermia treatments, but the presented methods can be easily applied to different biological targets.

The paper is organized as follows: The data acquisition method is explained in Section 2.1. The preparation of the input data and the output labeling for the DL model is given in Section 2.2. The architecture of the DL model is explained in Section 2.3, and the training parameters and performance are given in Section 2.4. The results are discussed in Section 3, and the paper ends with a conclusion in Section 4. It is also worth noting that $e^{-j\omega t}$ time dependency is used throughout the paper.

## Method

The proposed study has a patient-specific approach, in which it is assumed that a reference DP distribution of the breast cancer patient is available before the treatment process thanks to imaging modalities such as MRI and computed tomography (CT) [11, 12, 19]. Computer simulations for the data acquisition of random temperature distributions are performed to train the DL model offline. Once a proper model is obtained, it can be used during MH treatment to detect the hotspots arisen in the imaging domain.

### Data acquisition

In order to train the network, we need random temperature distributions and corresponding differential scattered field data. The differential data indicates the difference between the scattered field measurements between hot (heated) and cold (normal) breasts. In a practical scenario, both quantities can be measured with the help of calibration processes. However, it is not possible to use experimental data for training in this type of problem even if we were in a practical application because the training data must also include dangerous temperature levels, which is not safe for patients. Therefore, the training data must be generated by computer simulations.

**Fig. 1:**
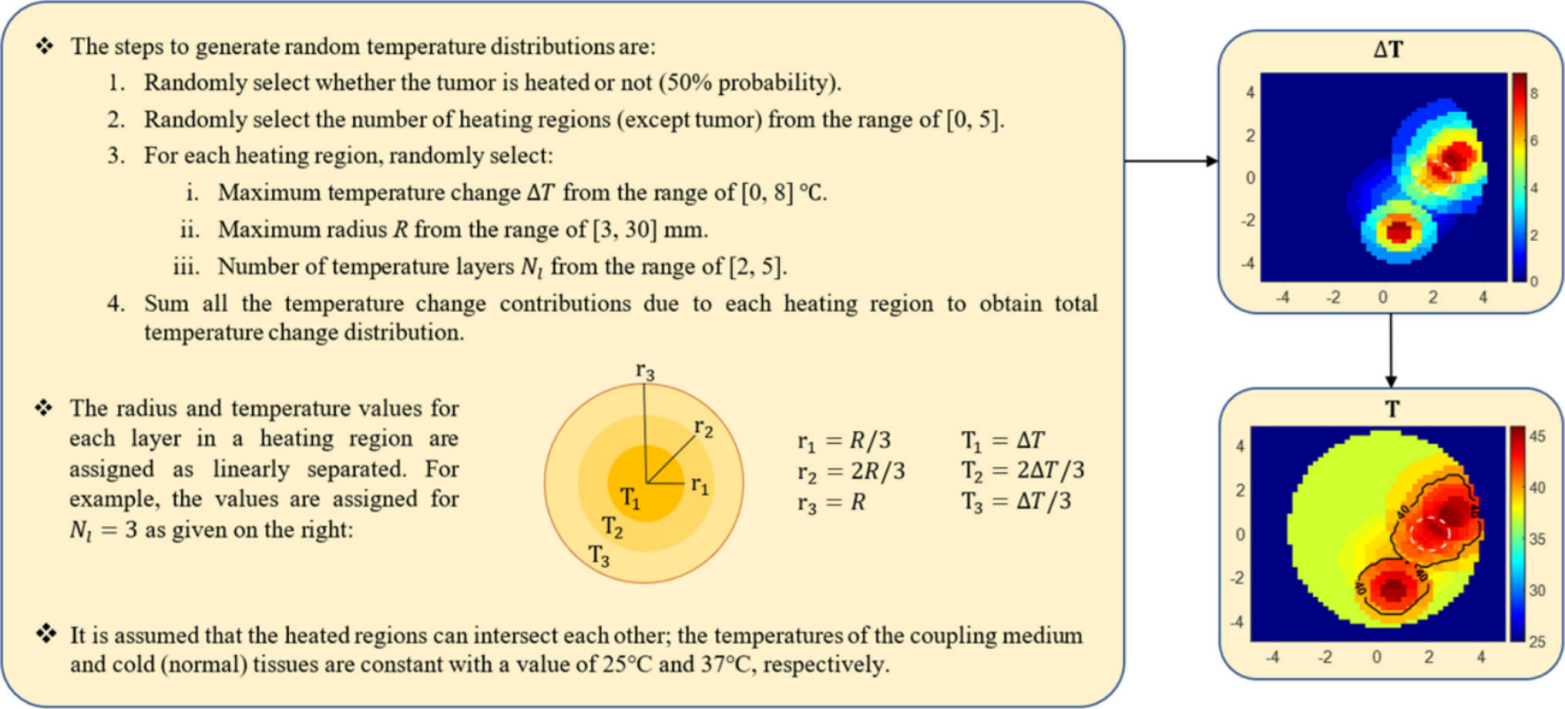
The procedure to generate a random temperature distribution (heating scenario). The dashed white circle encloses the tumor. Axes in cm. Temperatures in $^\circ$C

The key step in data acquisition is to generate random temperature distributions. One way to do this is by solving Pennes’ bioheat equation [22] numerically for random phase and amplitude values of the illuminating antennas; however, this type of solver code was not available for us during the study. Another way may be the use of commercial 3-D electromagnetic-thermal simulators; however, this process is intractable because 3-D solvers are computationally costly and a problem like in our study requires a large amount of data. Therefore, we have used another way, in which the heating studies for hyperthermia in the literature [23–29] are examined to get an insight into the behavior of the temperature distribution. Based on these studies, some important inferences can be made:

- The temperature distributions have smooth variations over the computation domain. This is expected because, according to Pennes’ bioheat equation [22], the second derivative of temperature with respect to the spatial coordinates must be continuous.
- Temperature values gradually decrease from the focal point (heating center) to the periphery of the heating region. Based on this, we define heating regions with layered geometry, in which the temperature decreases while moving away from the heating center.
- Multiple heating regions can arise over the whole treatment domain [25, 27, 29]. Therefore, the total number of heating regions is also selected randomly, and these regions are distributed over the domain.
- It is possible that the heating system can focus energy on regions except the tumor due to possible discrepancies between the simulation and practical application stages [29], even if the phase and amplitude parameters are well-determined at the simulation stage. Therefore, we also add the probability of whether the tumor is heated or not.

The details of the random temperature distribution procedure can be seen in Fig. 1.

It is assumed that the MRI-derived DP distributions of the patient are available before the treatment process [11, 12, 19]. The breast model used in this study is also MRI-derived [30]. These DP distributions are assumed to be reference distributions, which means the distributions at normal temperature (or cold case). The spatial resolution of the model used in the study is 2 mm. A circular tumor inclusion with a radius of 7.5 mm [31] is placed at (20, 0) mm location. The DPs of the tumor have been adopted from [32]. To find the DPs corresponding to the temperature distributions (hot cases), we must define a relationship between DPs and temperature. It is known that DPs of tissues are dependent on temperature, but the literature on temperature dependencies of DPs of real breast tissues is limited. However, there are papers giving a model for the temperature dependencies of animal tissues, such as bovine and porcine [20, 21]. Based on these works, it can be said that the relative permittivity ($\epsilon _{r}$) of the tissues decreases around 0.25% per 1 $^\circ$C increment, and effective conductivity ($\sigma$) increases around 1.25% per 1 $^\circ$C increment:

1 $$\begin{aligned} \Delta \epsilon _{r}(r,\omega ) = -\epsilon _{r}(r,\omega ) \left( 0.0025 \Delta T\right) , \end{aligned}$$

2 $$\begin{aligned} \Delta \sigma (r,\omega ) = \sigma (r,\omega ) \left( 0.0125 \Delta T\right) . \end{aligned}$$

We note that more details about the temperature dependency model can be found in [11].

**Fig. 2:**
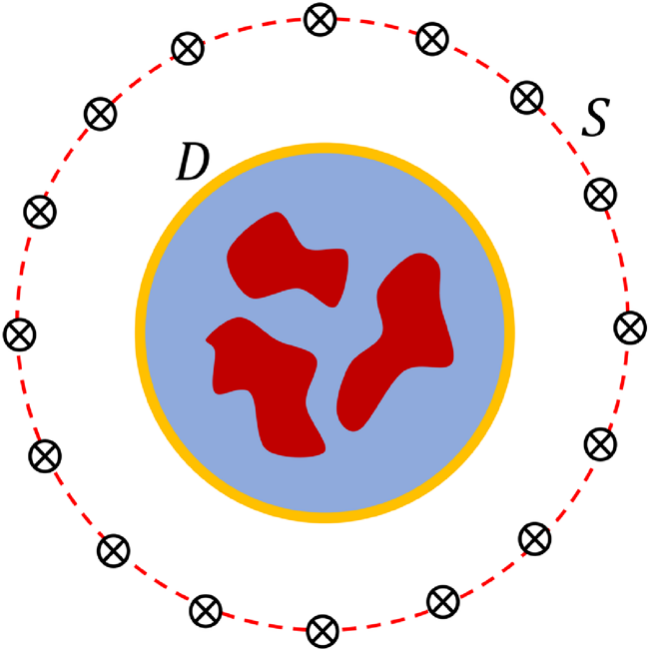
The general geometry of 2-D electromagnetic scattering problem with transverse magnetic (TM) polarization. The cross-shaped circles indicate the transmit-receive antennas. The *D* is the computation domain, and the *S* is the measurement domain

Once a temperature distribution and its corresponding DPs are determined, the direct scattering solver can be used to determine scattered electric fields. In this study, the problem is 2-D with transverse magnetic (TM) polarization, and the well-known method of moments (MoM)-based integral equation solver scheme presented in [33] is used as a direct solver. In an electromagnetic scattering problem, the data equation is given as follows:

3 $$\begin{aligned} E_{s}(r_{r}, r_{t},\omega ) = k_{0}^2(\omega ) \int _{D} G(r_{r}, r,\omega ) \chi (r,\omega )E(r, r_{t},\omega ) \,dr, \quad r_{r},r_{t} \in S, \ r \in D \end{aligned}$$

Here, $\chi$ denotes the contrast function given as

4 $$\begin{aligned} \chi (r,\omega ) = \epsilon _{r}^{c}(r,\omega ) - \epsilon _{rb}^{c}(\omega ) \end{aligned}$$

in which $\epsilon _{r}^{c}$ denotes complex relative permittivity defined as $\epsilon _{r}^{c}=\epsilon _{r}+j\sigma /\omega \epsilon _{0}$ where $\epsilon _{r}$ and $\sigma$ indicate relative permittivity and effective conductivity, respectively. The $\epsilon _{rb}^{c}$ is the complex relative permittivity of the coupling medium. Based on our previous work [11], relative permittivity and effective conductivity were selected as 20 and 0.6, respectively. Note that $\omega$ denotes angular frequency. Green’s function, which links the points in the computation domain *D* to the points in the measurement domain *S*, is given as $G(r_{r}, r, \omega ) = \frac{i}{4} H_{0}^{(1)}(k_b|r-r_{r}|)$. The $k_{0}=\omega /c$ and $k_{b}=(\omega ^{2}\epsilon _{r}\epsilon _{0}\mu _{0}+j\omega \sigma \mu _0)^{-1/2}$ are wavenumbers in free-space and coupling medium, respectively. For a known $\chi$, the total field *E* in computation domain *D* can be found by the MoM [33]. Then, the scattered field $E_{s}$ is computed by Eq. 3. It should be noted that *r*, $r_{r}$, and $r_{t}$ denote the locations of the points in the computation domain (or imaging domain) *D*, receiver antennas in the measurement domain *S*, and transmitter antennas in the measurement domain *S*, respectively. The general problem geometry is given in Fig. 2.

**Fig. 3:**
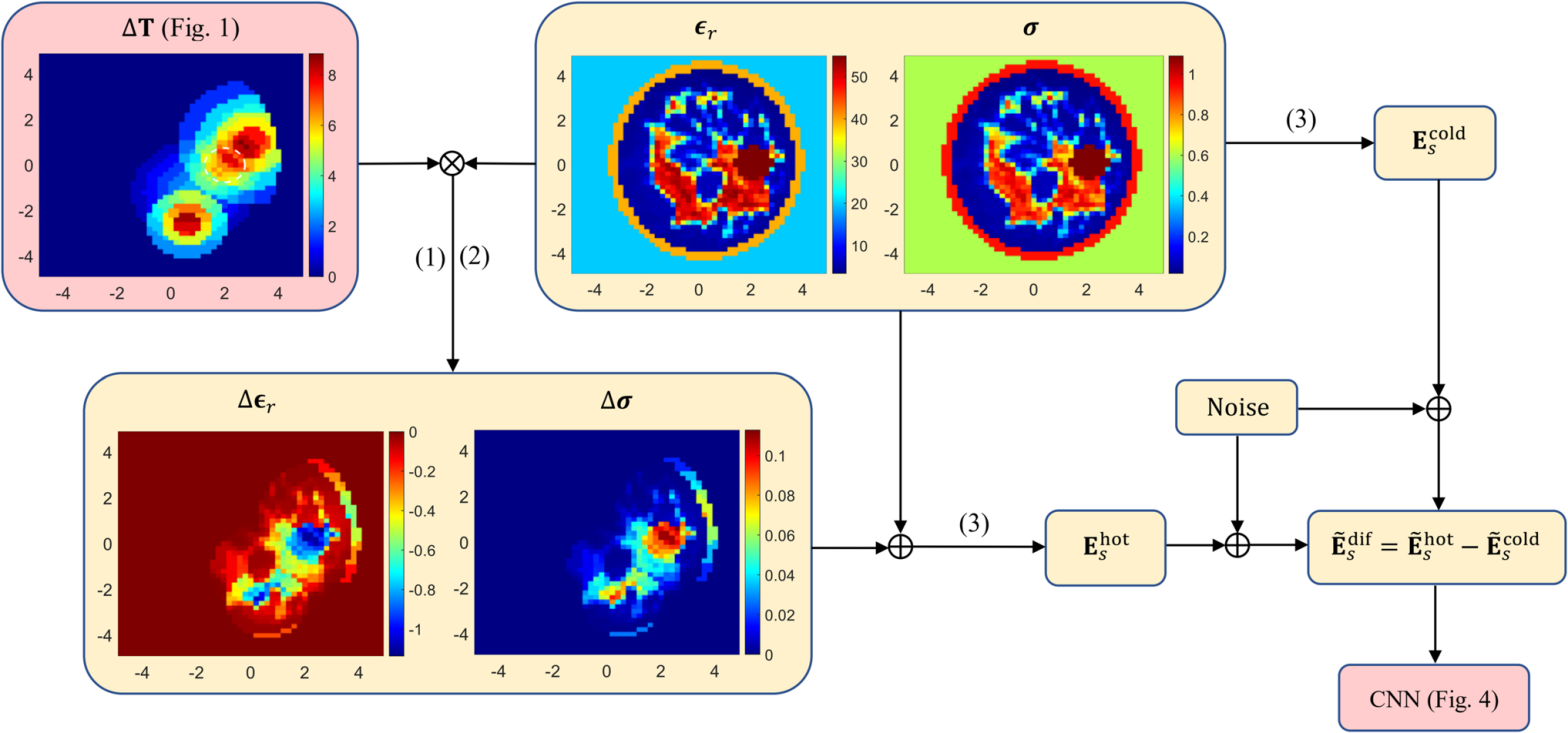
Summary of the data acquisition process. The numbers in parentheses refer to the equation numbers in the paper. Axes in cm. Temperatures in $^\circ$C

**Fig. 4:**
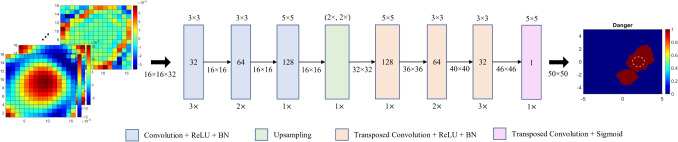
Architecture of the CNN model. The dashed white circle encloses the tumor. Axes in cm

The computation domain is illuminated by the antennas settled on a circle with a radius of 75 mm and modelled as line sources with an amplitude of 1. Multi-static measurement configuration is assumed, which means that while an antenna acts as a transmitter, other antennas act as receivers. The number of antennas is fixed at 16, which is a practically acceptable number [14]. Therefore, the scattered field $E_{s}$ is a 16$\times$16 matrix for a measurement at a specific frequency. It is assumed that the measurements are repeated for 16 different frequency points, which are linearly separated from the range of 0.5–2 GHz, which is a typical frequency interval for temperature monitoring applications [14]. Therefore, the scattered field data are complex-valued three-dimensional arrays with a size of 16$\times$16$\times$16. It is worth noting that we used multi-frequency data since we have experienced that the performance of the model has increased.

The data acquisition process for scattered field data can be summarized as follows: Firstly, the direct problem is solved once for the cold breast at each frequency to obtain the $\mathbf{{E}}_{s}^{\text {cold}}$. Secondly, a random temperature distribution is generated, and corresponding DPs are found. The direct problem is solved for these DP distributions to find the $\mathbf{{E}}_{s}^{\text {hot}}$. The second process is repeated to generate sufficient data for the DL network. The overall process is summarized in Fig. 3.

### Data preparation

The input data are differences between scattered electric fields from the hot and cold states of the breast. Additive white Gaussian noise (AWGN) is also added to both the hot and cold data separately. The signal-to-noise ratio (SNR) is defined as $10\text {log}_{10}(P_{\text {E}_s}/P_{\text {Noise}})$, in which $P_{\text {E}_s}$ and $P_{\text {Noise}}$ are the power of the scattered electric field and the noise signal. The SNR for the training and test data are denoted by $\text {SNR}_\text {train}$ and $\text {SNR}_\text {test}$, respectively. The reason for the noise in the test data is to mimic practical measurement. The noise in the training data is added for two reasons: First, the MRI-derived DPs of the patient will have some error; therefore, simulated scattered field data will also contain some level of noise. Second, adding noise to the training data is a method to increase the generalization performance of the neural network [34]. We have experienced that training with noisy data has remarkable regularization effects, which can greatly increase the performance of the model.

Another process for the input data is to separate real and imaginary parts since the electric field data consist of complex numbers. For this purpose, real and imaginary parts at a specific frequency are concatenated along the third axis of the array. By this way, the input data at a specific frequency is 16$\times$16$\times$2 in size. The same process is applied to the data at each frequency, and these are also concatenated in the same way. As a result, the final form of the input data size becomes 16$\times$16$\times$32.

The output of the DL model (grand truth label) is an image with a size of 50$\times$50, in which each pixel takes values of 0 or 1. The dangerous temperature level for healthy tissues is accepted as 40 $^\circ$C [35] in this study. The zero indicates that the temperature of the pixel is smaller than 40 $^\circ$C, and the one indicates that the temperature of the pixel is greater than 40 $^\circ$C (hotspot). Therefore, the output of the model is a hotspot distribution over the domain. Therefore, the problem can be considered a pixel-wise binary classification problem. An example image is at the end of the flowchart given in Fig. 4.

### Architecture of the model

The architecture of the model is given in Fig. 4. This is an encoder-decoder structure, of which some variants have been utilized for the solution of ISPs. We select a CNN structure because its performance is shown by some studies in the literature [15–18]. We fixed the number of antennas, number of frequencies, and the number of data for the training, and the model complexity is gradually increased and hyperparameters are tuned to enhance the performance of the model. The structure is constructed almost symmetrically. In the blocks given in Fig. 4, the number inside the block indicates the number of output filters. The number at the top of the blocks (except the upsampling block) is kernel size, and the bottom of the blocks is the repeating number of the same layer. All the convolutional layers apply zero padding to keep same the output size, but transposed convolution layers do not apply any padding to increase the size of the output to match the final output image with a size of 50$\times$50. Notice that we have also used an upsampling layer to double the size of the output in both dimensions. Each convolution and transposed convolution layers except the output layer apply rectified linear unit (ReLU) activation function and batch normalization (BN). The output layer includes sigmoid function as activation function. We did not perform any pooling operation to avoid loss of information.

**Fig. 5:**
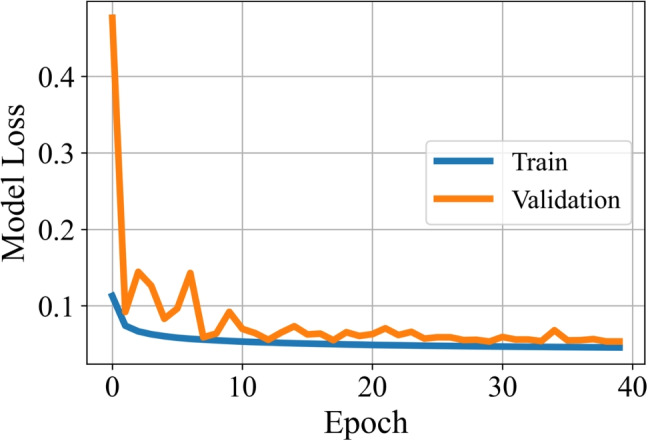
The variation of model loss with respect to epoch

**Fig. 6:**
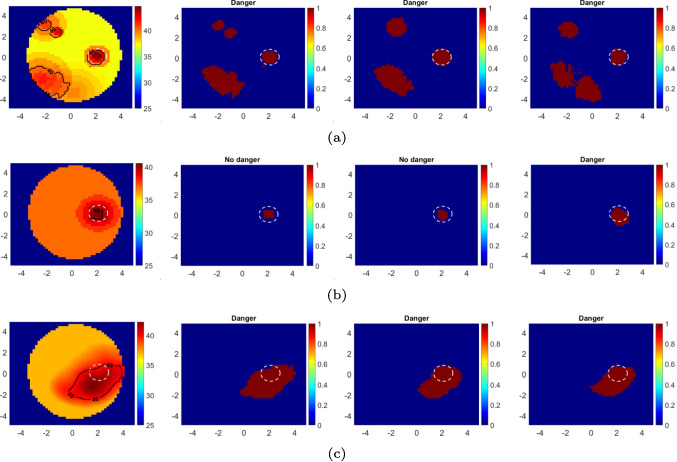
First column: True temperature distributions ($\textbf{T}$) in $^\circ$C. Second column: True images showing the hotspot distributions. The estimated hotspot distributions by the proposed CNN model for $\text {SNR}_\text {test}=40$ dB (Third column) and $\text {SNR}_\text {test}=30$ dB (Fourth column). The results were obtained for $\text {SNR}_\text {train}=40$ dB. Notice that the danger status indicates that there is at least one hotspot outside the tumor region. The dashed white circle encloses the tumor. Axes in cm

### Training

The training was performed using TensorFlow 2.10 and Keras API. The total number of data is 50,000, which is divided into three sets with a ratio of 75%, 15%, and 15% for training, validation, and testing, respectively. Then, the number of training data is 37,500, and validation and test data are 7500. The binary cross-entropy loss function is selected as the cost function. We use stochastic gradient descent (SGD) with a momentum of 0.8 as the optimization method. Exponential decay is preferred as a learning rate scheduler, in which the learning rate is determined as $\eta (t)=\eta _{0}r^{t/v}$ at step *t*. The initial learning rate ($\eta _{0}$) is 0.01, and the decay rate (*r*) is 0.1. The learning rate decays at every *v* steps, where $v= \left\lfloor {N_{\text {epoch}} N_{\text {train}}/S_{\text {batch}}}\right\rfloor$. The batch size ($S_{\text {batch}}$) is 32, and the number of epochs ($N_{\text {epoch}}$) is 40. The $N_{\text {train}}$ denotes the number of training data, and $\text {SNR}_\text {train}=40$ dB.

The variation of the loss for training and validation is given in Fig. 5. It can be said that the validation loss is smoothly converging to the training loss, which is an indicator of a good learning process. On the other hand, the final training and validation accuracies are 0.981 and 0.978, respectively. The test accuracies are calculated as 0.978 for $\text {SNR}_\text {test}=40$ dB and 0.968 for $\text {SNR}_\text {test}=30$ dB.

**Fig. 7:**
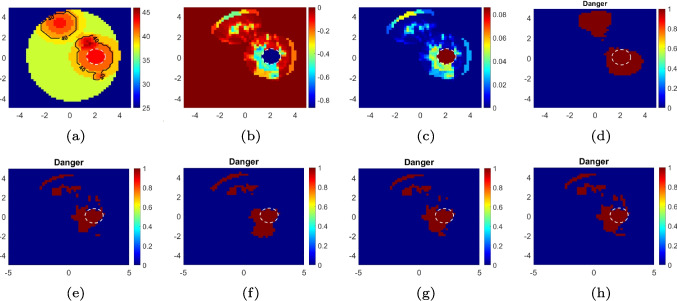
A sample temperature distribution in $^\circ$C (**a**) and corresponding changes in relative permittivity (**b**) and effective conductivity (S/m) (**c**). The estimated hotspot distributions by **d** the proposed CNN model, and **e**–**h** the method presented in [11] combined with TR, TSVD, CGLS, and FISTA, respectively. Note that $\text {SNR}_\text {test}$ and $\text {SNR}_\text {train}$ are 40 dB. The dashed white circle encloses the tumor. Axes in cm

## Results and discussion

The results for some of the test data are given in Fig. 6, which show that the proposed model can accurately estimate hotspots in the imaging domain. Further, to quantitatively measure the performance of the proposed model, the accuracy score is defined as follows:

5 $$\begin{aligned} s_{\text {acc}} = \frac{TP+TN}{TP+TN+FP+FN} \end{aligned}$$

The *TP*, *FP*, *TN*, and *FN* are true positive, false positive, true negative, and false negative counts, respectively. The average values of $s_{\text {acc}}$ ($\overline{s}_{\text {acc}}$) for the test data are 0.959 and 0.939 for $\text {SNR}_\text {test}=40$ dB and $\text {SNR}_\text {test}=30$ dB, respectively. It should be noted that the temperature distribution given in the last row of Fig. 6 was obtained by electromagnetic-thermal simulation in CST Studio software. These results verify that the trained model also works with real temperature distributions, which means that the proposed data acquisition approach is acceptable and that the model learns the underlying physics of the problem.

In order to see the success of the proposed model more clearly, it is compared with conventional inversion methods. We have used the method presented in [11], in which the BIM is combined with a regularization technique to estimate the temperature distribution over the imaging domain. Four different well-known regularization techniques—Tikhonov regularization (TR), truncated singular value decomposition (TSVD), conjugate gradient least squares (CGLS), and the fast iterative shrinkage-thresholding algorithm (FISTA)—were selected for this study. The results for a sample temperature distribution are given in Fig. 7. As it was examined in detail in [11], the conventional regularization methods cannot accurately estimate temperature changes on the tissues with low DPs. This is because these changes correspond to very small changes in the DPs of these tissues, and it is reasonable to think that these small contributions are filtered by these regularization methods. However, the result given in Fig. 7d verifies that the proposed model does not suffer from this issue. This is an indicator of the strong regularization properties of the data-driven methods. To quantify this, we additionally use the Dice score, which is defined as follows:

6 $$\begin{aligned} s_{\text {Dice}} = \frac{2TP}{2TP+FP+FN}. \end{aligned}$$

The Dice score ($s_{\text {Dice}}$) measures the proportion of overlap between the two images. The Dice score is useful to quantify the performance of the methods aspect from the issue related to the small DP changes. This is because the Dice score focuses on the TP count, which indicates the number of pixels whose temperature actually exceeds the threshold value and is correctly detected. The average accuracy and Dice scores are calculated using each method for 100 random test data. The results are given in Table 1, which shows that the proposed CNN model is superior to other regularization techniques.

**Table 1:** The average accuracy and Dice scores for 100 test data

Method	CNN	TR	TSVD	CGLS	FISTA
$\overline{s}_{\text {acc}}$	0.932	0.889	0.882	0.891	0.894
$\overline{s}_{\text {Dice}}$	0.671	0.420	0.420	0.446	0.451

**Fig. 8:**
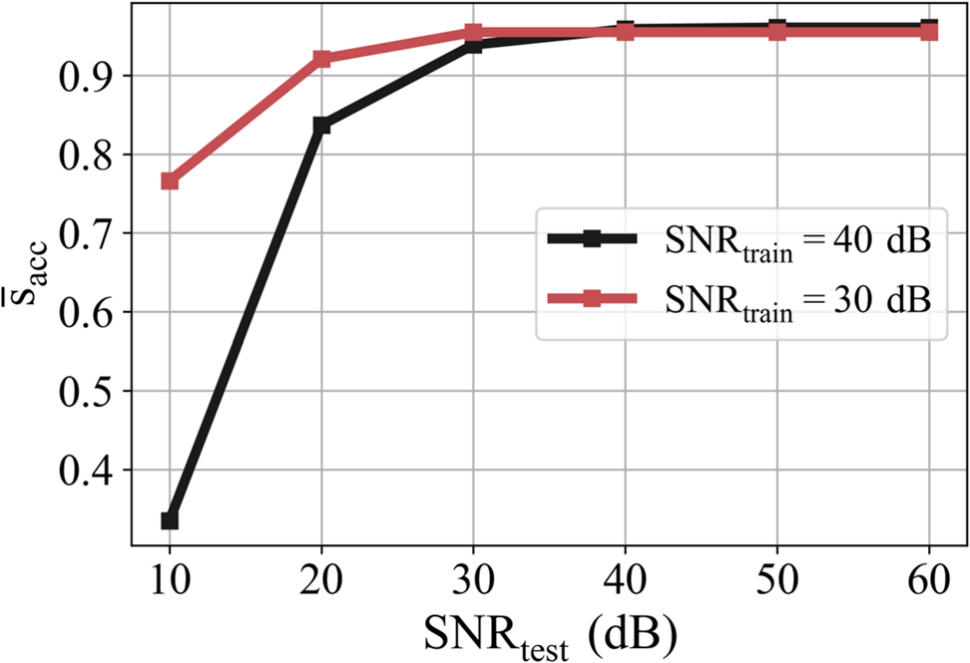
The variation of the average accuracy score ($\overline{s}_{\text {acc}}$) with respect to the SNR in the test data ($\text {SNR}_\text {test}$)

The effect of noise is especially important for this type of problem because input data values are very small since the temperature dependency of the DPs of the tissues is very small. To investigate this topic, average accuracy scores ($\overline{s}_{\text {acc}}$) are computed for the test data with different $\text {SNR}_\text {test}$ values. The result is given in Fig. 8, which shows that when $\text {SNR}_\text {test}$ is close to or greater than $\text {SNR}_\text {train}$, the performance of the model is preserved. From a practical point of view, this shows that it may be important to approximately know the noise level experienced by the measurement system. This prior knowledge can be considered a restriction in a practical application; however, the results show that it may have a critical effect on the performance of the model.

It should be noted that the most important parameter for patient safety is the number of false negatives, which indicates that there is at least one cell exceeding the dangerous temperature level, but the model cannot detect it. Therefore, we use another metric to measure the detection performance of the method. Remember that our model estimates a hotspot distribution as an image. Without focusing on this overall distribution, we can only focus on whether there is a hotspot in a region outside the tumor. This is because if there is a hotspot inside the tumor region, this does not indicate a danger since it is generally desirable to heat the tumor as much as possible. We define a danger accuracy score as follows:

7 $$\begin{aligned} s_{\text {acc}}^{\text {danger}} = \frac{TP_{\text {danger}}}{TP_{\text {danger}}+FN_{\text {danger}}} \end{aligned}$$

**Fig. 9:**
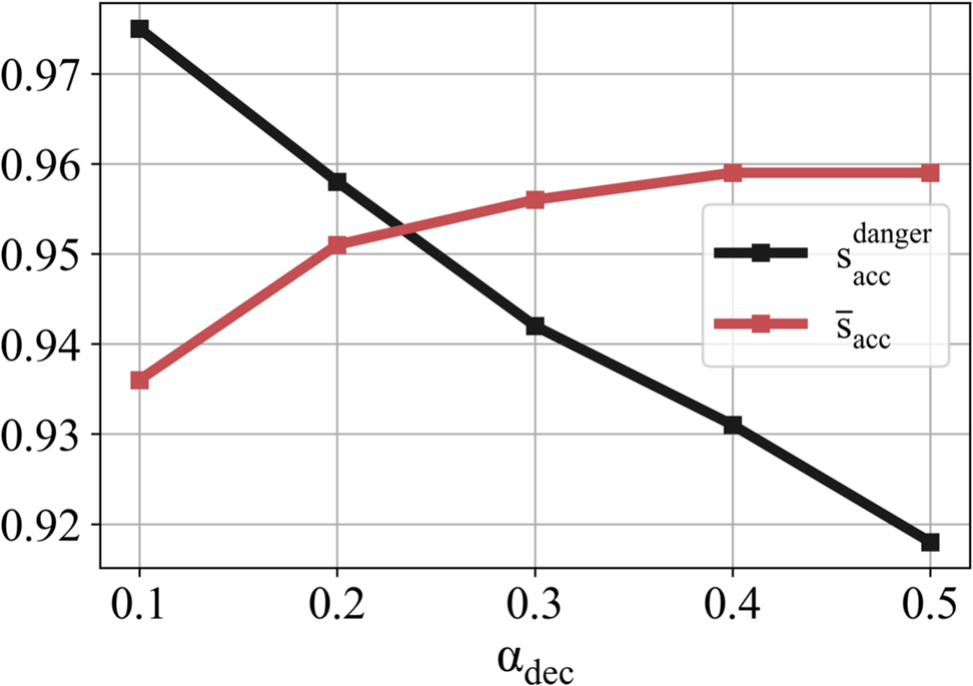
The variations of the average accuracy score ($\overline{s}_{\text {acc}}$) and the danger accuracy score ($s_{\text {acc}}^{\text {danger}}$) with respect to decision threshold ($\alpha _{\text {dec}}$). Note that $\text {SNR}_\text {test}$ and $\text {SNR}_\text {train}$ are 40 dB

The danger accuracy ($s_{\text {acc}}^{\text {danger}}$) for $\text {SNR}_\text {test}=40$ dB is 0.918. Moreover, $s_{\text {acc}}^{\text {danger}}$ can be increased by decreasing the decision threshold $\alpha _{\text {dec}}$, which indicates the threshold to determine the class of an output of the CNN model. In other words, if the probability value of an output of the model is greater than $\alpha _{\text {dec}}$, it is assumed to be a hotspot. Until this point, $\alpha _{\text {dec}}$ was 0.5. To see the effect of $\alpha _{\text {dec}}$, the variation of the $s_{\text {acc}}^{\text {danger}}$ with respect to $\alpha _{\text {dec}}$ is given in Fig. 9. It is observed that $s_{\text {acc}}^{\text {danger}}$ can be increased up to around 0.98. However, there is a trade-off between $\overline{s}_{\text {acc}}$ and $s_{\text {acc}}^{\text {danger}}$, as expected.

**Fig. 10:**
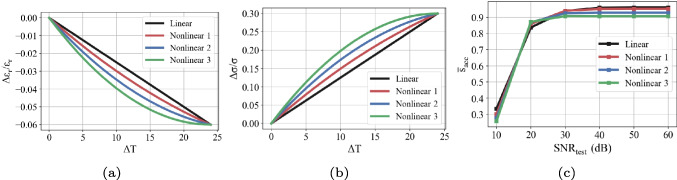
The relative changes in **a** relative permittivity and **b** effective conductivity (S/m) with respect to change in temperature for different degrees of nonlinearity. **c** The accuracy scores for these different nonlinear models. Note that $\text {SNR}_\text {train}$ is 40 dB

The relative changes in DPs are assumed to vary linearly with temperature. Even if this assumption is not bad according to the studies in the literature [20, 21], the actual relation is slightly nonlinear. Therefore, it may be important to investigate how the performance of the proposed method varies depending on the degree of nonlinearity. This indicates the case where training and testing operations are performed under the linear model assumption as done before, but the actual model is nonlinear. The curves for different degrees of nonlinearity are given in Fig. 10 a and b. The nonlinear model is represented by a quadratic function. The model is given for the range of 0–24 $^\circ$C since the maximum temperature change value in the test data is 24. The results for the accuracy scores are given in Fig. 10c. According to the results, the accuracy values are above 0.9 in all cases when $\text {SNR}_\text {test}$ is greater than 30 dB. This shows that the proposed method maintains its performance even at a high degree of nonlinearity.

Finally, it might be useful to elaborate on how the proposed methodology could be applied in a clinical setting. When the patient presents to the hospital for treatment, the DP distributions of the patient’s breast at room temperature can be determined by imaging modalities such as MRI and CT. These reference DPs are used for data generation, and the parameters of the proposed DL model are determined specifically for the patient. After that, the model is ready to be used for hotspot prediction. In microwave imaging systems, antennas and vector network analyzers (VNA) are used for S-parameter measurement. The model presented in this paper uses electric field values as input data. Therefore, the transition from the measured S-parameter to the electric field should be done using calibration methods. (Differently, if an analytical relationship between the *S*-parameter and the electric field can be established in the system used, the DL model can be trained with the *S*-parameter as input values.) Once the hyperthermia system starts the heating process, it takes measurements for imaging at regular intervals. During the treatment, the medical staff can monitor the images obtained from these measurements and get information about whether the heating process is proceeding correctly. Furthermore, the system can automatically shut itself down in a dangerous situation, preventing harm to the patient.

## Conclusion

In this paper, a CNN model is proposed to detect hotspots during microwave hyperthermia (MH) treatments. The problem can be considered a multi-output binary classification problem, in which the outputs indicate pixels in the image. The data are generated using an in-house data generator, which mimics the possible temperature distribution in a MH heating process. The model is also tested with real temperature distributions obtained from commercial simulation software. It can be said that the results reveal three main conclusions: First, the data-driven methods have stunning performance for hotspot detection problem. Second, the data-driven methods have very different and strong regularization properties when compared with conventional regularization methods. Finally, training with noise may be crucial especially for hyperthermia monitoring problem. A future study may focus on the combination of deep learning and conventional inversion methods to further increase accuracy in the MH treatment monitoring problems.
